# Hypercapnic respiratory failure with insufficient response to fixed-level PS-NIV: Is AVAPS the end solution?

**DOI:** 10.5578/tt.20239925

**Published:** 2023-06-13

**Authors:** D. Bhattacharya, G. A. Mohanchandra, M. MANDAL, A. M. ESQUINAS

**Affiliations:** 1 Department of Anesthesiology, R. G. Kar Medical College and Hospital, Kolkata, India; 2 Department of Anesthesiology, Institute of Post Graduate Medical Education and Research, Kolkata, India; 2 Intensive Care Unit, Hospital Morales, Meseguer, Murcia, Spain

To the Editor,

We read with great interest the study of Acet Öztürk, et al. ( 1 ) where they observed that average volume-assured pressure support (AVAPS) mode can be beneficial over fixed level pressure support (PS) during non-invasive ventilation (NIV) therapy in patients with acute hypercapnic respiratory failure (AHRF). We appreciate the author’s observation that AVAPS-NIV can reach the target tidal volume and help reduce PaCO2 as compared to PS-NIV ( 1 ). However, we raise certain points for further clarification and a better understanding of their reports.

First, N-terminal pro-B-type natriuretic peptide (NT-pro BNP) is found to be increased in congestive heart failure (CHF) and hypervolemia. Monitoring of this biomarker is important in predicting the outcome in hypercapnic respiratory failure patients who often have comorbidity such as heart failure. The cut-off value of NT-pro BNP with no greater than 1.000 pg/mL has a high sensitivity (95%) and a negative likelihood ratio (0.09) during spontaneous breathing trials and can indicate weaning failure ( 2 ).

Second, body positioning can influence ventilation. In semirecumbent or lateral positions there is a significant reduction in PaCO2 value ( 3 ). We are curious to know whether the authors have utilized the advantages of body posture and NT-pro BNP monitoring in their management.

Third, the severity of COPD (FEV1/FVC< 0.7 and FEV1< 80%) and history of previous hospitalization are also important predictors of outcome ( 4 ). A considerable proportion of the population in the Western as well as in the developing countries suffers from obesity ( 5 ).

Fourth, the results of this study would have been further interesting if the authors could have included a greater number of ”current smokers“ (4 out of 35 in the present study) in their study. Smokers are prone to developing acute hypoxemic respiratory failure (AHRF) in the context of chronic obstructive pulmonary disease (COPD), and hypercapnia tends to be less responsive to improvement with NIV. It would also be interesting to analyze whether using AVAPS-NIV as a first-line therapy could be beneficial in this population.

Fifth, it would have been more useful if this study clearly outlined the correlation between the use of AVAPS-NIV and improvement in APACHE II scores with respect to FiO2 requirement, respiratory rate, and arterial pH. Since higher APACHE II scores are a determinant of NIV failure, we wonder if AVAPS-NIV use influenced any reduction in APACHE II scores ( 6 )

Sixth, AVAPS-NIV has been demonstrated to decrease the total duration of NIV usage in AHRF. In this context, establishing an objective definition of prolonged hospital stay would be beneficial in providing a clearer assessment of the advantages of AVAPS-NIV.

Seventh, AVAPS has been reported to be safe and reliable in the management of stable hypercapnic COPD patients and found to be advantageous compared with fixed-level PS-NIV in terms of better perception of sleep efficiency and quality of life (QoL) ( 7 ). We are curious to know if the authors have assessed the improvement in sleep quality and QoL as well. Furthermore, we encourage further studies with larger sample sizes to strengthen and consolidate the findings.
